# IETA Ultrasonic Features Combined with GI-RADS Classification System and Tumor Biomarkers for Surveillance of Endometrial Carcinoma: An Innovative Study

**DOI:** 10.3390/cancers14225631

**Published:** 2022-11-16

**Authors:** Dongmei Lin, Hui Wang, Lu Liu, Liang Zhao, Jing Chen, Hongyan Tian, Lei Gao, Beibei Wu, Jing Zhang, Xia Guo, Yi Hao

**Affiliations:** 1 Department of Medical Ultrasonics, South China Hospital of Shenzhen University, Shenzhen 518000, China; 2 Department of Medical Ultrasonics, The Seventh Affiliated Hospital of Sun Yat-sen University, Shenzhen 518000, China; 3 Department of Medical Ultrasonics, Dongguan People’s Hospital, Dongguan 523000, China; 4 Shenzhen Key Laboratory of Viral Oncology, Center for Clinical Research and Innovation (CCRI), Shenzhen Hospital, Southern Medical University, Shenzhen 518000, China

**Keywords:** the international endometrial tumor analysis (IETA), gynecologic imaging reporting and data system (GI-RADS), tumor biomarkers, endometrial cancer/carcinoma (EC), endometrium lesion, uterine cavity lesions

## Abstract

**Simple Summary:**

Endometrial cancer (EC) is one of the most common malignant tumors in gynecology. The prognosis of patients with early EC is good; however, high-risk EC patients have a poor prognosis because they have a higher risk of tumor recurrence, lymph node metastasis, and distant tumor spread. At present, there are no structured ultrasound reporting standards for endometrial lesions. The high specificity of serum tumor biomarkers for EC has not been found in current studies. The present results show that the international endometrial tumor analysis (IETA) ultrasonic features combined with gynecologic imaging reporting and data system (GI-RADS) and tumor biomarkers provides a novel, safe, real-time technology for surveillance of EC. This study is clinically significant since it shows that the IETA ultrasonic features combined with the GI-RADS classification system and tumor biomarkers method have good performance in discriminating EC. It has the potential to be used as a screening tool to distinguish the benign and malignant lesions of the uterine cavity or endometrium very well.

**Abstract:**

*Objectives*: We were the first to combine IETA ultrasonic features with GI-RADS and tumor biomarkers for the surveillance of endometrial carcinoma. The aim was to evaluate the efficacy of single IETA ultrasonography GI-RADS classification and combined tumor biomarkers in differentiating benign and malignant lesions in the uterine cavity and endometrium. *Methods:* A total of 497 patients with intrauterine and endometrial lesions who had been treated surgically between January 2017 and December 2021 were enrolled; all of them had undergone ultrasound examinations before surgery. We analyzed the correlation between the terms of ultrasonic signs of the uterine cavity and endometrial lesions defined by the expert consensus of IETA and the benign and malignant lesions and then classified these ultrasonic signs by GI-RADS. In addition, the tumor biomarkers CA125, CA15-3, CA19-9 and HE4 were combined by adjusting the classification. The results of the comprehensive analysis were compared with pathological results to analyze their diagnostic efficacy. *Results:* (1) The statistic analysis confirmed that there were seven independent predictors of malignant lesions, including thickened endometrium (premenopause ≥ 18.5 mm, postmenopause ≥ 15.5 mm), non-uniform endometrial echogenicity (heterogeneous with irregular cysts), endometrial midline appearance (not defined), the endometrial–myometrial junction (interrupted or not defined), intracavitary fluid (ground glass or “mixed” echogenicity), color score (3~4 points) and vascular pattern (focal origin multiple vessels or multifocal origin multiple vessels). (2) In traditional ultrasound GI-RADS (U-T-GI-RADS), if category 4a was taken as the cut-off value of benign and malignant, the diagnostic sensitivity, specificity, PPV, NPV and diagnostic accuracy were 97.2%, 65.2%, 44.0%, 98.8% and 72.2%, respectively, and the area under the ROC curve (AUC) was 0.812. If 4b was taken as the cut-off value, the diagnostic sensitivity, specificity, PPV, NPV diagnostic accuracy and AUC were 88.1%, 92.0%, 75.6%, 96.5% and 91.2%, 0.900, respectively. The diagnostic sensitivity, specificity, PPV, NPV diagnostic accuracy and AUC were 75.2%, 98.5%, 93.2%, 93.4%, 93.4% and 0.868, respectively, when taking category 5 as the cutoff point. In modified ultrasound GI-RADS (U-M-GI-RADS), if 4a was taken as the cut-off value, The diagnostic efficacy was the same as U-T-GI-RADS. If 4b was taken as the cut-off value, the diagnostic sensitivity, specificity, PPV, NPV, diagnostic accuracy and AUC were 88.1%, 92.3%, 76.2%, 96.5%, 91.3% and 0.902, respectively. If 4c was taken as the cutoff point, the diagnostic sensitivity, specificity, PPV, NPV diagnostic accuracy and AUC were 75.2%, 98.7%, 94.3%, 93.4%, 93.6% and 0.870, respectively. The diagnostic sensitivity, specificity, PPV, NPV diagnostic accuracy and AUC were 66.1%, 99.7%, 98.6%, 91.3%, 92.4% and 0.829, respectively, if taking category 5 as the cutoff point. (3) In the comprehensive diagnostic method of U-T-GI-RADS combined tumor biomarkers results, the AUC of class 4a, 4b and 5 as the cutoff value was 0.877, 0.888 and 0.738, respectively. The AUC of class 4a, 4b, 4c and 5 as the cutoff value in the comprehensive diagnostic method of U-M-GI-RADS combined tumor biomarkers results was 0.877, 0.888, 0.851 and 0.725, respectively. There was no significant difference in diagnostic efficiency between the two comprehensive diagnostic methods. *Conclusions:* In this study, no matter which diagnostic method was used, the best cutoff value for predicting malignant EC was ≥GI-RADS 4b. The GI-RADS classification had good performance in discriminating EC. The tumor biomarkers, CA125, CA19-9, CA15-3 and HE4, could improve the diagnostic efficacy for preoperative endometrial carcinoma assessment.

## 1. Introduction

Endometrial cancer is one of the three most common malignant tumors in the female reproductive system, which seriously threatens the life and health of women [1,2,3,4,5]. It comprises about 20–30% of gynecological malignancies [6]. Patients with early-stage endometrial cancer have a good prognosis. If treated promptly, the 5-year survival rate of stage IA patients is > 90% [7,8,9]. However, high-risk endometrial cancer patients have a poor prognosis because they have a higher risk of tumor recurrence, lymph node metastasis, and distant tumor spread [7,9,10]. Therefore, early detection and characterization of uterine or endometrial lesions for appropriate management are critical. The adequate identification of malignant and benign lesions is the first and foremost step for correct and optimal treatment [11]. Thus far, ultrasound is the preferred method for suspected ovarian tumors [11]; the same holds true in the uterine cavity and endometrial lesions. The transvaginal ultrasonography examination is atraumatic, cheap, well tolerated and can be widely used in clinical practice [12], but ultrasound varies in its ability to determine whether a lesion is malignant, and precise diagnosis depends on technical skill and experience [13].

A tactic to provide a unified and structured language for ultrasound reporting of gynecological tumors is clearly required. It is proposed that a structured ultrasound reporting system would be even more beneficial if it had proper clinical utility and improved communication between radiologists and clinicians [14]. By far, ultrasound medical techniques have already built up a number of guidelines and structured reports to characterize AM (adnexal masses), including simple grading systems, subjective assessments and statistically derived scoring systems [15].

The GI-RADS was first created by Amor et al., in 2009, as a way to implement structured reporting for AMs. The system is based on the recognition patterns and criteria proposed by the International Ovarian Cancer Analysis (IOTA) [16]. The system nominates six categories of malignancy, ranging from normal to high-risk malignancy. It has been successfully prospectively and externally validated [17]. The IETA consistent nomenclature and definitions [9,10,18] for all characteristics of tumors assessed by ultrasound has ameliorated discrimination of uterine cavity or endometrial lesions by including quantitative assessment of morphological features. However, there were only a few studies [19,20,21,22,23,24,25,26,27,28] on the application of these expert consensuses, and it has not been widely promoted in China. Moreover, at present, there is no structured ultrasound reporting standard for a uterine cavity or endometrial lesions. The inconsistent diagnostic level and description terms in these areas contributed to confusion in the interpretation of ultrasound reports by clinicians, and it was not conducive to the development of the professional level in this field [29].

The high specificity of serum tumor biomarkers for endometrial cancer has not been found in current studies [3,30,31]. CA125 was important in the diagnosis and follow-up of female reproductive system tumors [32]; it is usually observably enhanced in the vast majority of patients with ovarian tumors. However, it may also be elevated to varying degrees in patients with benign lesions or healthy people. HE4 (human epididymal protein 4) has recently been recommended as an underlying biomarker for malignant endometrial lesions [3,4,33,34]. Previous studies demonstrated that HE4 and CA125 were observably correlated with myometrial invasion, cervical infiltration, lymph node metastasis and histological grade and stage [3,5,35,36,37,38,39]. CA19-9 is closely relevant to gastrointestinal neoplasms and gynecologic neoplasms. CA15-3 is a significant and particular tumor biomarker for breast carcinoma [40]. It has also been discovered to be highly expressed in malignant lesions of the digestive tract and endometrium in the last few years [41]. Some studies found that examination of imaging jointed tumor biomarker detection can significantly improve the ability of tumor diagnosis [6,39,40,42]. However, there were few literatures that reported on the comprehensive evaluation of endometrium or uterine cavity lesions by using IETA ultrasound features combined with GI-RADS or tumor biomarkers.

In this research, we proposed to adopt IETA ultrasound characteristics and GI-RADS to decrease the effect of subjective judgment on ultrasound examination results of the uterine cavity or endometrial lesions. We normalized the description of IETA ultrasonic features and classified them into GI-RADS classification system according to the IETA expert consensus literature [9,10,18], the IOTA simple standard method, the criteria used for GI-RADS adnexal tumors classification [43], the previous research experience of our project team and the correlation between ultrasound features defined by IETA and benign and malignant lesions. The grade of GI-RADS is GI-RADS 1-5, and the higher the grade, the more likely the lesion is malignant [44]. Then, the classification was adjusted in combination with the results of CA125, CA15-3, CA19-9 and HE4. The results of the comprehensive analysis were compared with pathological results to analyze its diagnostic efficacy.

The main objective of the comprehensive evaluation system for intrauterine and endometrial lesions is to diagnose or rule out malignancy, reduce unnecessary surgery in benign lesions and optimize the prognosis of endometrial cancer by timely referral to a gynecologic oncologist in malignant lesions.

## 2. Materials and Methods

Four hundred and ninety-seven patients with benign or malignant lesions of the endometrium or uterine cavity who underwent ultrasound and tumor biomarker examination and surgical treatment at The Seventh Affiliated Hospital of Sun Yat-sen University (414 cases) or Shenzhen Hospital of Southern Medical University (76 cases) or South China Hospital of Shenzhen University (this hospital is a newly built one; it didn’t officially open for full services until 30 June 2021; therefore, only 7 cases were included) between January 2017 and December 2021 were enrolled in the study. The retrospective study was approved by the Medical Ethics Committee of the three hospitals (KY-2020-030-01, NYSZYYEC20200029 and 20211126001), and the need for informed consent was waived.

### 2.1. The Inclusion Criteria for This Research Were as Shown Below

(1) Tumor biomarkers HE4, CA125, CA15-3 and CA19-9 were detected in all cases within 1 month before the operation;
(2) All cases underwent transrectal/transvaginal ultrasonography within 1 month before the operation;
(3) The patients underwent human chorionic gonadotropin (HCG) testing to rule out pregnancy-related diseases;
(4) Adenomyosis was not observed in all cases, and the abnormalities of the endometrium–myometrium junction caused by adenomyosis were excluded;
(5) The enrolled patients underwent hysteroscopy, curettage or surgical resection, and the results were confirmed by pathological diagnosis or surgical records.

### 2.2. The Exclusion Criteria Were as Follows

(1) All cases that did not meet the inclusion criteria;
(2) The patients had previously undergone lower abdominal surgery, and the uterus had been excised;
(3) Any patient who was allergic to ultrasound gel;
(4) Patients who were not of legal age (under 18 years) were not included for medical ethical reasons;
(5) The patients who have received preoperative hormone therapy, chemotherapy, radiation therapy or tumors in other organs;
(6) The patients who have recently taken hormone drugs or pregnant or lactating women.

### 2.3. Instruments and Methods

In this study, we used 3D ultrasound instruments (GE Voluson E10 and E8 GE Healthcare, Tiefenbach, Zipf, Austria). All instruments were equipped with high-frequency intracavity probes (RIC5-9-D, 5–9 MHz). All patients needed to empty the bladder, take the lithotomy position and undergo a transvaginal (or transrectal) ultrasound scan, combined with a transabdominal ultrasound scan when necessary.

Every evaluation of the uterus should begin with a distinction between the bladder and cervix. The position of the uterus was recorded and measured. The uterus was scanned in the (oblique) transverse plane from the fundus to the cervix and in the sagittal plane from the uterine horn to the uterine horn. After establishing a panoramic view of the entire uterus, the image was enlarged to include only the body of the uterus. The magnification should be as large as possible and focused on the area of interest [18]. The ultrasound images of the uterus and bilateral appendages were collected from left to right in multiple sections of the probe, including gray-scale images, color Doppler images and power Doppler images. The images or dynamic videos were stored in the ultrasound workstation or machine hard disk.

### 2.4. The Theoretical Basis of IETA Ultrasonic Features GI-RADS Classification

In this study, we normalized the description of IETA ultrasonic features and classified them into GI-RADS classification system according to the IETA expert consensus literatures [9,10,18], the IOTA simple standard method, the criteria used for GI-RADS adnexal tumors classification [43], the previous research experience of our project team [29] and the correlation between ultrasound features defined by IETA and benign and malignant lesions. The objective was to use standard descriptions to help sonographers classify intrauterine and endometrial lesions to distinguish between benign and malignant lesions. 

### 2.5. The Benign IETA Ultrasonographic Signs (B-Signs) Were as Follows

(1) Endometrial thickness: ≤4.0 mm (LR− < 0.1);
(2) Uniform endometrial echogenicity: homogeneous hyperechoic, homogeneous hypoechoic, homogeneous isoechoic, three-layer pattern;
(3) Non-uniform endometrial echogenicity: homogeneous with regular cysts;
(4) Endometrial midline appearance: linear;
(5) Endometrial–myometrial junction: regular;
(6) “Bright edge”: yes;
(7) Color score: 1~2 points;
(8) Vascular pattern: no flow, single vessel (without branching), circular vessels.

### 2.6. The Malignant IETA Ultrasonographic Signs (M-Signs) Were as Follows

(1) Endometrial thickness: premenopause ≥ 18.5 mm (LR+ > 10), postmenopause ≥ 15.5 mm (LR+ > 10);
(2) Non-uniform endometrial echogenicity: heterogeneous with irregular cysts;
(3) Endometrial midline appearance: not defined;
(4) Endometrial–myometrial junction: interrupted, not defined;
(5) Intracavitary fluid: ground glass, “mixed” echogenicity;
(6) Color score: 3~4 points;
(7) Vascular pattern: multiple vessels (focal origin), multiple vessels (multifocal origin).

### 2.7. The Undefined IETA Ultrasonographic Signs (U-Signs) Were as Follows

(1) Non-uniform endometrial echogenicity: homogeneous with irregular cysts;
(2) heterogeneous without cysts; heterogeneous with regular cysts;
(3) Endometrial midline appearance: non-linear, irregular;
(4) Endometrial–myometrial junction: irregular;
(5) “Bright edge”: no;
(6) Intracavitary fluid: no fluid; anechoic or of low-level echogenicity;
(7) Vascular pattern: single vessel (with branching), scattered vessels.

**Figure 1:**
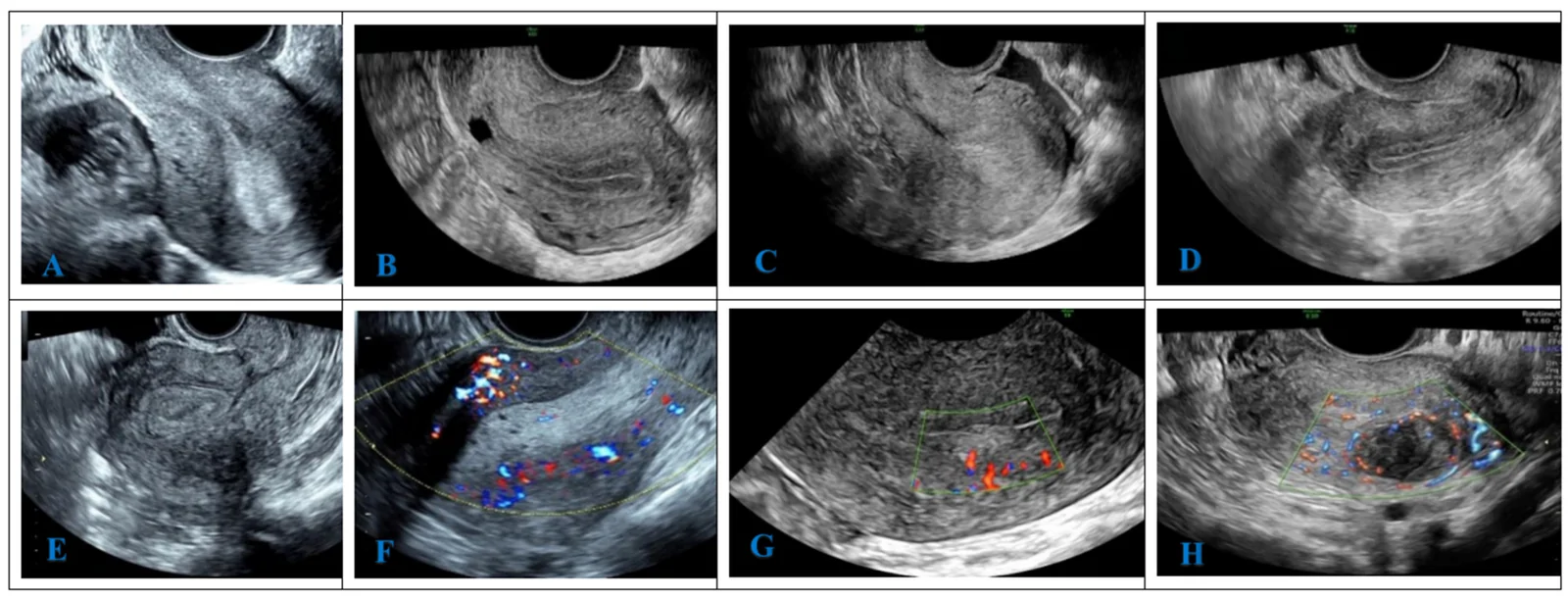
Images of benign IETA ultrasonographic features. (**A**) Homogeneous hyperechoic; (**B**) Three-layer pattern; (**C**) Homogeneous isoechoic; (**D**) Endometrial midline appearance: “linear” and endometrial–myometrial junction: “regular”; (**E**) The “bright edge” sign, the echo formed by the interface between an intracavitary lesion and the endometrium; (**F**) Homogeneous with regular cysts; (**G**) Vascular pattern: single vessel (without branching); (**H**) Vascular pattern: circular vessels.

**Figure 2:**
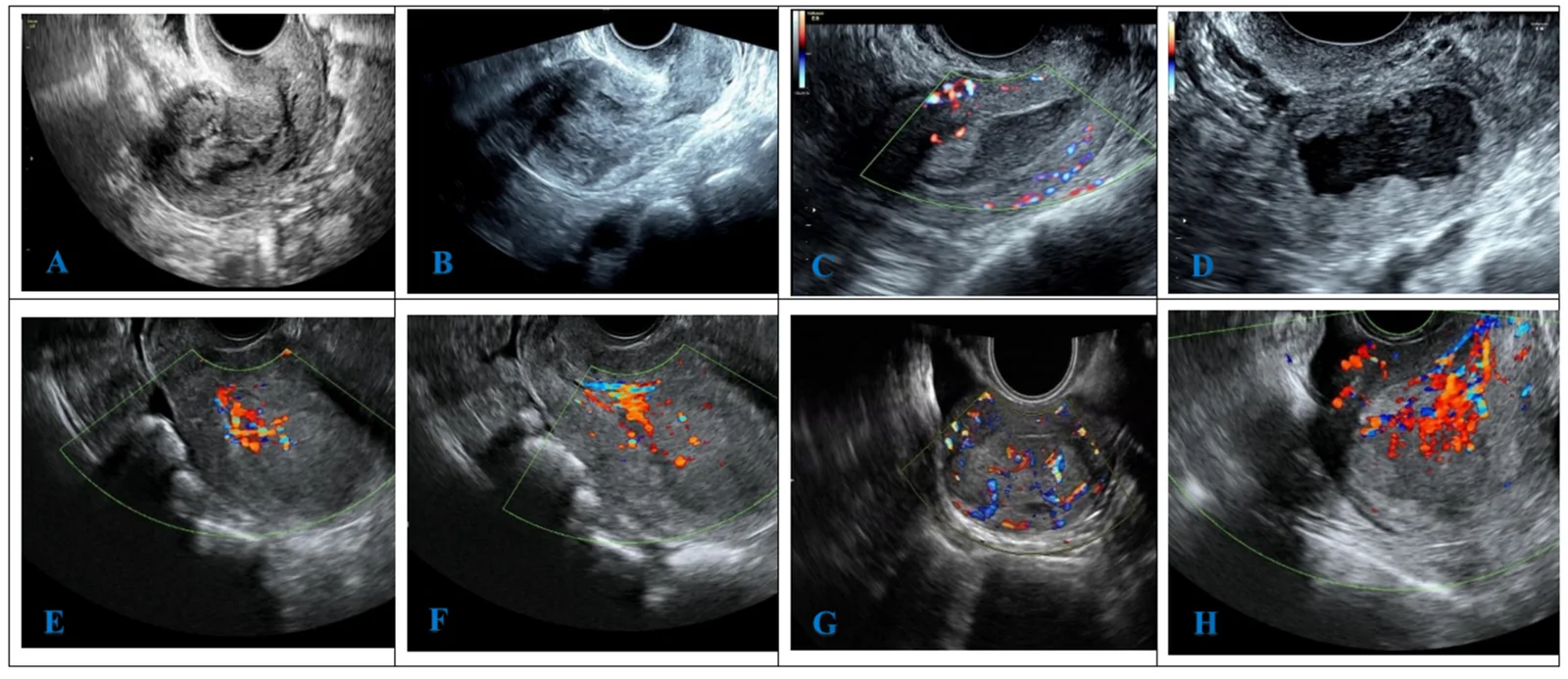
Images of malignant IETA ultrasonographic features. (**A**) Heterogeneous with irregular cysts and endometrial–myometrial junction: “interrupted”; (**B**) Endometrial midline appearance: “not defined” and endometrial–myometrial junction: “not defined”; (**C**) Intracavitary fluid: “ground glass”; (**D**) Intracavitary fluid: “mixed” echogenicity; (**E**,**F**) Vascular pattern: Multiple vessels (focal origin); (**G**) Vascular pattern: Multiple vessels (multifocal origin); Color score: moderate amount of color/moderate vascularity (3 points); (**H**) Color score: abundant color/abundant vascularity (4 points).

See Table 1, Table 2, Table 3 and Table 4 and Figure 1 and Figure 2 for details.

### 2.8. Ultrasonic Image Analysis

The ultrasound images of all enrolled cases were randomly sorted (the research coordinator coded the stored images to hide the patients’ personal information). Two senior sonographers with more than 10 years of experience in gynecological ultrasound independently analyzed all ultrasound images with no additional information after fully understanding the IETA expert consensus on the specific content of endometrial or uterine cavity lesions. All lesions were graded by GI-RADS classification and compared with pathological results to evaluate their diagnostic efficacy. 

### 2.9. The Serological Detection of Tumor Markers CA125, CA15-3, CA19-9 and HE4

Five milliliters of venous blood from the patient was used as a test sample and centrifuged for 15 min at 3000 r/min. The serum tumor biomarkers, HE4, CA125, CA15-3 and CA19-9, were detected by chemiluminescence. The detection instrument was an Abbott I-2000 chemiluminescence instrument; the detection reagents were the product of the original factory. 

The normal reference value range: HE4: premenopause < 70 pmol/L, post-menopause < 140 pmol/L; CA l9-9 ≤ 35 U/mL; CA l5-3 ≤ 35 U/mL; CA l25 ≤ 35 U/mL.

The abnormal evaluation criteria: Tumor biomarker measurements exceeding normal values were considered positive.

### 2.10. The Comprehensive Evaluation

The intrauterine or endometrial lesions were classified by the GI-RADS classification method based on IETA ultrasonography and then evaluated these lesions comprehensively by combining the results of serum tumor biomarkers CA125, CA15-3, CA19-9 and HE4. When there are two or more tumor biomarkers reading higher than the reference value in the ultrasonic GI-RADS category 3–5 lesions, the lesions should be upgraded, for example, from 4a to 4b. When one tumor biomarker was elevated, and the other three tumor biomarkers were normal, the lesion level did not rise or fall. When the values of the four tumor biomarkers were all within the normal range, the lesions were downgraded, such as from class 4a to class 3. The sensitivity, specificity, positive predictive value (PPV), negative predictive value (NPV), diagnostic accuracy, area under ROC curve (AUC) and 95% confidence interval (95% CI ) of this classification were calculated. 

### 2.11. Statistical Analysis

SPSS version 23.0 software (IBM Corp. Released 2015. IBM SPSS Statistics for Windows, Version 23.0. Armonk, NY: IBM Corp., USA) was used for all statistical analyses in this study. The quantitative data were expressed as mean ± standard deviation, and homogeneity of variance and normal distribution were tested. The nonparametric test or independent-samples *T*-test was used for the quantitative data. The percentage (%) was used to express the value of categorical variables. The Chi-square test or Fisher’s exact test was used for classification variables data comparison.

The Chi-square test and binary logistic regression were used for univariate and multivariate analysis. With pathological results or surgical records (cases of intrauterine adhesions) as the gold standard, the ROC curve was used to analyze the sensitivity, specificity and coincidence rate of simple IETA ultrasonographic features GI-RADS classification method and combined tumor biomarker results from comprehensive analysis for benign and malignant diagnosis of the uterine cavity and endometrial lesions. 

The AUC value (1.0–0.5) was used to evaluate the diagnostic effect. When AUC = 0.5, it indicated that the diagnostic method was completely invalid and had no diagnostic value. When AUC > 0.5, the closer the AUC was to 1, the better the diagnostic effect was. When the AUC was between 0.5 and 0.7, the diagnostic value was low. The AUC was between 0.7 and 0.9, and the diagnostic value was medium. The AUC > 0.9 had a high diagnostic value. *P* value <0.05 was considered statistically significant.

## 3. Results

### 3.1. General Information

A total of 497 patients were included in the study. The patients were 19–86 (41.58 ± 11.44) years old. The cases were classified as a benign group and a malignant group according to the surgical records or pathological findings. (Because the current clinical management of endometrial hyperplasia with atypia was based on the reference to malignant lesions, these cases were classified as malignant lesions in this study).

There were 388 benign lesions, including 181 endometrial polyps, 47 endometrial simple hyperplasia, 11 endometrial complex hyperplasia, 56 endometrial polypoid hyperplasia, 35 submucous myomas, 8 cases of endometritis and 50 cases of intrauterine adhesions. There were 109 malignant lesions, including 88 cases of endometrioid adenocarcinoma (FIGO stage IA: 57, IB: 8, II: 13, IIIA: 1, IIIC: 6, IV: 3), 9 cases of endometrial atypical hyperplasia, 1 case of endometrial low-grade squamous intraepithelial lesion, 3 cases of uterine carcinosarcoma (FIGO stage IB: 1, IIIB: 1, IVA: 1), 1 case of giant cell type high-grade undifferentiated sarcoma of uterus, 1 case of poorly differentiated endometrioid adenocarcinoma with focal clear cell differentiation (FIGO stage IB), 1 case of endometrial carcinoma (FIGO stage IA, 50% endometrioid adenocarcinoma and 50% mucinous adenocarcinoma), 1 case of mixed endometrial carcinoma (FIGO stage IIIC2, endometrioid carcinoma and serous carcinoma), 1 case of serous adenocarcinoma of endometrium (FIGO stage IIIA), 1 case of endometrial infiltrating adenocarcinoma (FIGO stage IB, 80% were highly differentiated endometrioid adenocarcinoma and 20% mucinous adenocarcinoma), 1 case of endometrial clear cell carcinoma (FIGO stage IIIA ) and 1 case of endometrial undifferentiated carcinoma (FIGO stage II).

There were statistically significant differences in BMI, age, the proportion of patients before and after menopause, the number of pregnancies, the number of abortions, the proportion of patients with irregular vaginal bleeding, and the proportion of patients with contact bleeding or leucorrhea with blood (all *p* < 0.05). The clinical variables and demographic were shown in Table 5.

### 3.2. Univariate Analysis and Multivariate Logisitic Regression Analysis of IETA Ultrasonographic Features 

There were statistically significant differences in the signs between benign lesions and malignant lesions (all *p* < 0.05) among the nine groups of ultrasonic signs included in the study (Table 1).

The positive characteristics of the above univariate analysis were included in multivariate logistic regression analysis, and seven independent predictors of malignant lesions were obtained. They were thickened endometrium (premenopause ≥18.5 mm, postmenopause ≥15.5 mm), non-uniform endometrial echogenicity (heterogeneous with irregular cysts), endometrial midline appearance (not defined), endometrial–myometrial junction (interrupted or not defined), intracavitary fluid (ground glass or “mixed” echogenicity), color score (3~4 points), vascular pattern (multiple vessels (focal origin) or multiple vessels (multifocal origin)). The Exp(B) values were 1.279, 11.426, 7.435, 105.102, 53.333, 119.516 and 186.811, respectively (all *p* < 0.01). See Table 2 for detail.

**Figure 3:**
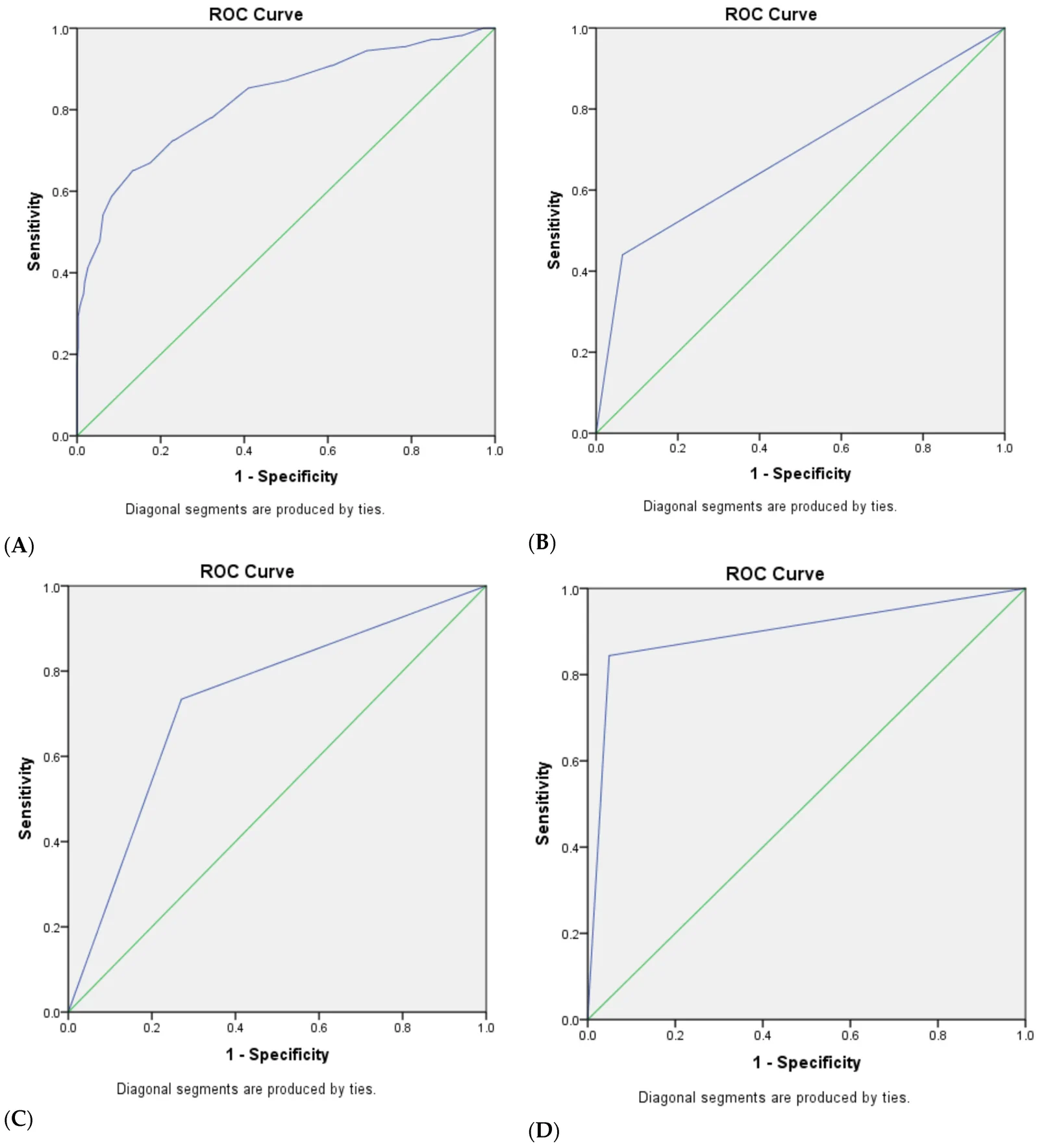

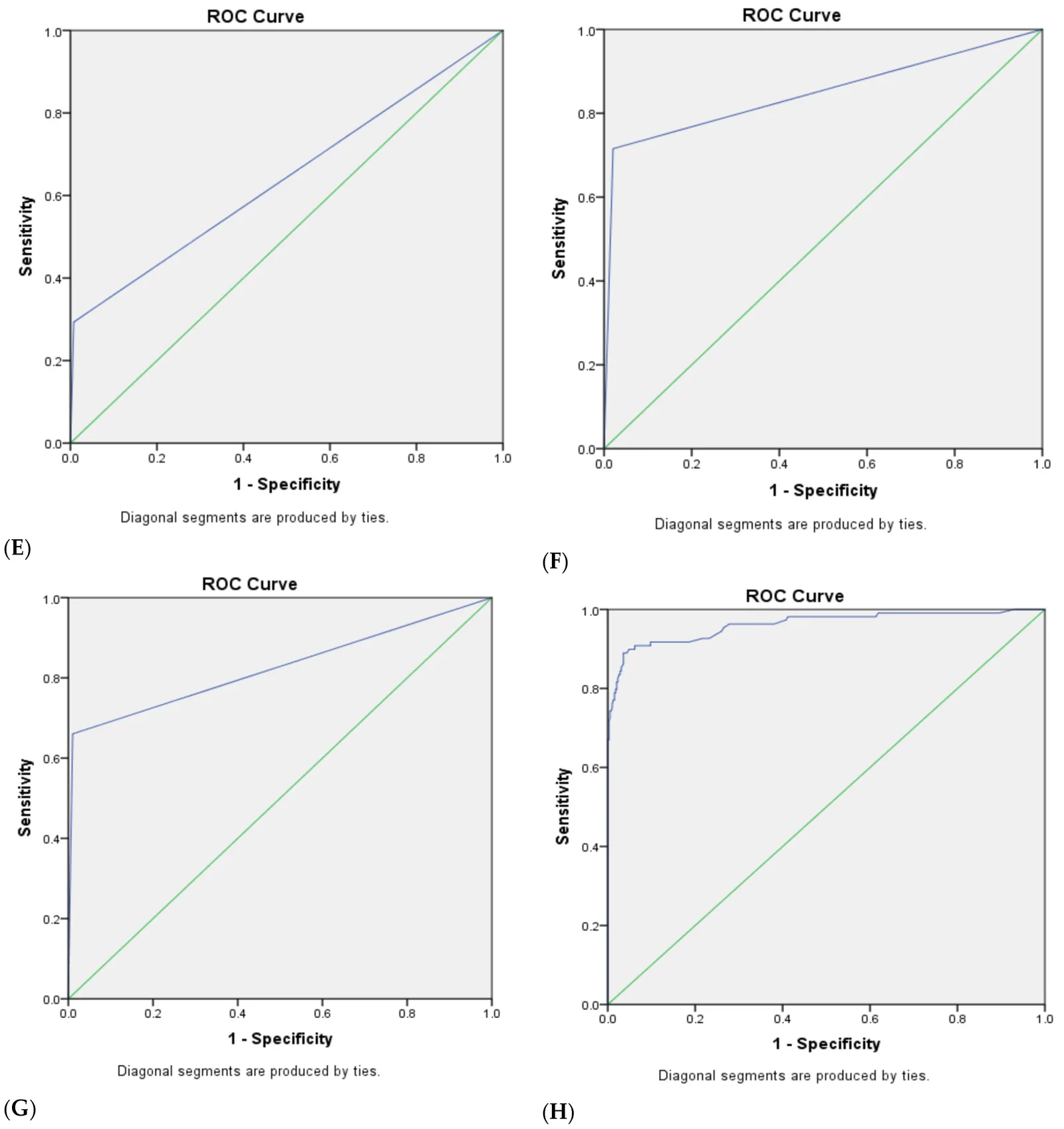
The ROC curves of various malignant ultrasound signs and their combination. (**A**) The ROC curve of endometrial thickness malignant signs; AUC: 0.826, SE: 0.025, 95% CI (0.777, 0.874). (**B**) The ROC curve of non-uniform endometrial echogenicity malignant signs “ heterogeneous with irregular cysts”; AUC: 0.688, SE: 0.032, 95% CI (0.625, 0.751). (**C**) The ROC curve of endometrial midline appearance malignant signs “not defined”; AUC: 0.732, SE: 0.028, 95% CI (0.677, 0.786). (**D**) The ROC curve of endometrial–myometrial junction malignant signs “interrupted or not defined”; AUC: 0.898, SE: 0.021, 95% CI (0.856, 0.939). (**E**) The ROC curve of intracavitary fluid malignant signs “ground glass or mixed echogenicity”; AUC: 0.643, SE: 0.033, 95% CI (0.577, 0.709). (**F**) The ROC curve of color score malignant signs “3~4 points”; AUC: 0.847, SE: 0.027, 95% CI (0.795, 0.900). (**G**) The ROC curve of vascular pattern malignant signs: multiple vessels (focal origin) or multiple vessels (multifocal origin); AUC: 0.825, SE: 0.028, 95% CI (0.770, 0.881). (**H**) The ROC curve of combination of multiple ultrasonic malignant signs; AUC: 0.962, SE: 0.012, 95% CI (0.938, 0.986).

The ROC curves were drawn to obtain the area under the curve (AUC) of various ultrasonic malignant signs, among which the AUC of the endometrial–myometrial junction (interrupted or not defined ) was the largest (0.898) and the AUC of the intracavitary fluid (ground glass or “mixed” echogenicity) was the smallest (0.643) (all *p* < 0.01). After the combination of these ultrasound features, the diagnostic efficiency was further improved, and the AUC could reach 0.962 (Shown in Supplementary Table S1 and Figure 3).

### 3.3. The Diagnostic Efficacy of Traditional and Modified Ultrasonic GI-RADS Classification in Predicting Benign and Malignant Uterine Cavity and Endometrial Lesions

With pathological results or surgical records as the “gold standard”, the traditional ultrasonic GI-RADS classification (U-T-GI-RADS) used grades 4a, 4b and 5 as the critical values, and the modified ultrasonic GI-RADS classification (U-M-GI-RADS) used grades 4a, 4b, 4c and 5 as the critical values, respectively. The sensitivity, specificity, positive predictive value (PPV), negative predictive value (NPV) and diagnostic accuracy (ACC) of the diagnosis of uterine and endometrial lesions are shown in Table 6. The diagnostic accuracy of the U-M-GI-RADS was slightly higher than that of U-T-GI-RADS, but the difference was not statistically significant. 

In U-T-GI-RADS, the area under the ROC curve (AUC) of class 4a, 4b and 5 as the cutoff value was 0.812 (95% CI: 0.774~0.850), 0.900 (95% CI: 0.862~0.939), 0.868 (95% CI: 0.819~0.918), respectively. The AUC of class 4a, 4b, 4c and 5 as the cutoff value in U-M-GI-RADS was 0.812 (95% CI: 0.774~0.850), 0.902 (95% CI: 0.863~0.940), 0.870 (95% CI: 0.820~0.919), 0.829 (95% CI: 0.774~0.884), respectively. There was no significant difference in diagnostic efficiency between the two ultrasonic GI-RADS diagnostic methods. With 4b as the truncation value, the diagnostic efficiency was the highest in the two diagnostic methods (Table S2).

### 3.4. The Combined Diagnostic Efficacy of Ultrasonic GI-RADS Classification Combined with Serum Tumor Biomarker (CA125, CA15-3, CA19-9 and HE4) Results for Benign and Malignant Uterine Cavity and Endometrial Lesions

**Figure 4:**
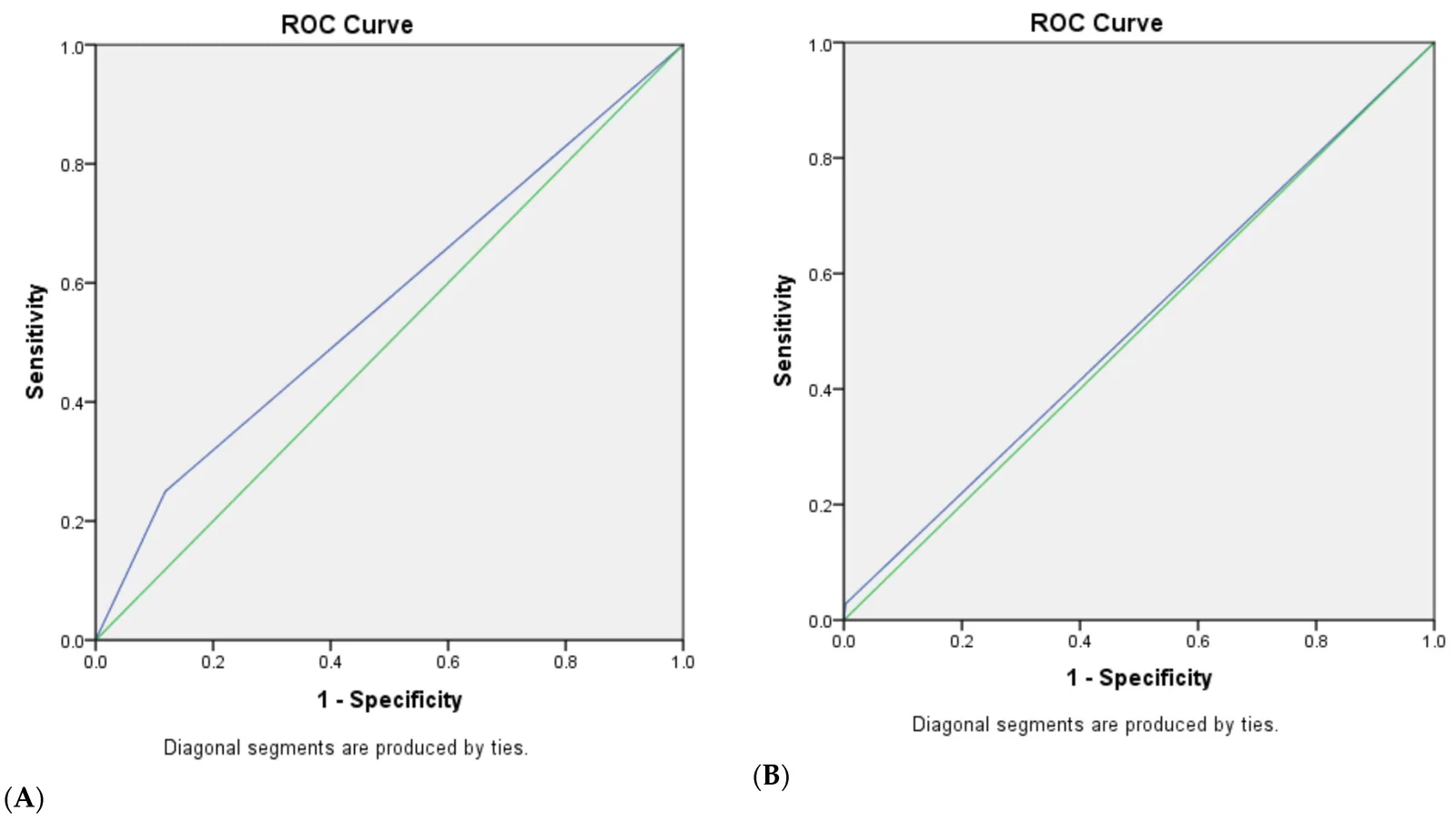

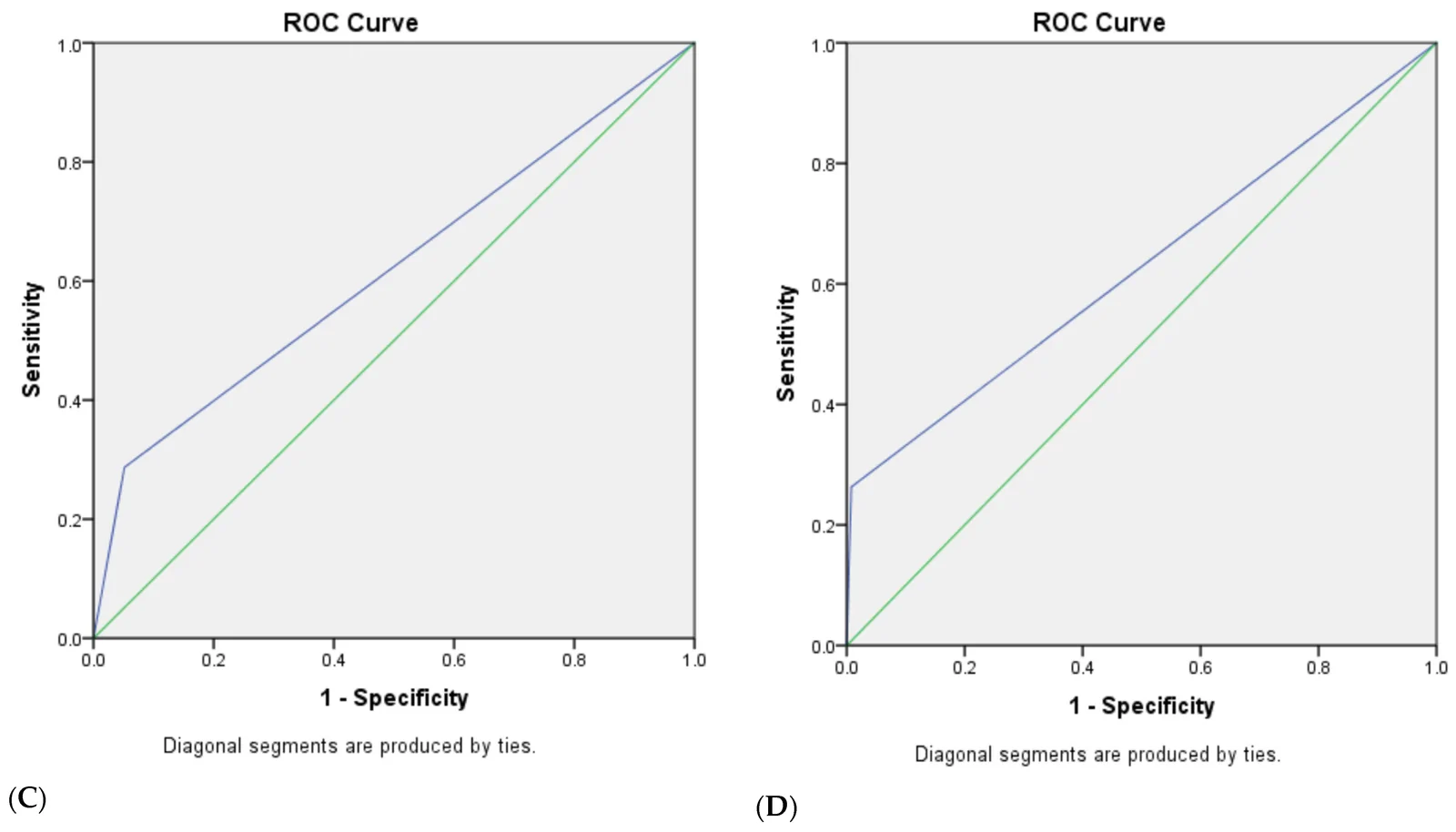
The ROC curve of serum tumor biomarkers. (**A**) The ROC curve of CA125 positive value; AUC: 0.566, SE: 0.033, 95% CI (0.502, 0.630). (**B**) The ROC curve of CA15-3 positive value; AUC: 0.513, SE: 0.032, 95% CI (0.450, 0.575). (**C**) The ROC curve of CA19-9 positive value; AUC: 0.618, SE: 0.033, 95% CI (0.553, 0.683). (**D**) The ROC curve of HE4 positive value; AUC: 0.628, SE: 0.036, 95% CI (0.558, 0.698).

As shown in Table S1 and Figure 4, the diagnostic efficacy of tumor biomarkers CA15-3 positive rate was low (AUC: 0.513); it showed no statistically significant difference in benign and malignant lesions (*p* = 0.689). The areas under the curve (AUC) of CA125, CA19-9 and HE4 positive rates were 0.566, 0.618 and 0.628, respectively. The difference between CA125, CA19-9 and HE4 positive rates in benign and malignant lesions was statistically significant (*p* values were 0.037, <0.01 and <0.01, respectively).

Among 497 enrolled cases, 33 cases (6.6%) were accompanied by at least two positive values of CA125, CA15-3, CA19-9 and HE4. Eight cases (2.1%) were benign lesions, including three cases of submucous myomas, four cases of endometrial polyps and one case of endometrial polypoid hyperplasia. Twenty-five cases (22.9%) were malignant, including twenty-one cases of endometrioid adenocarcinoma, one case of giant cell type high-grade undifferentiated sarcoma of the uterus, one case of endometrial clear cell carcinoma, one case of endometrial carcinoma (50% endometrioid adenocarcinoma and 50% mucinous adenocarcinoma) and one case of mixed endometrial carcinoma (endometrioid carcinoma and serous carcinoma). See Tables S3 and S4 for details of the results of different pathological types. The classification of benign lesions was mainly 1~4a, and that of malignant lesions was mainly 4a~5 (Table S5).

In the comprehensive diagnostic method of U-T-GI-RADS combined with tumor biomarkers results, the area under the ROC curve (AUC) of class 4a, 4b and 5 as the cutoff value was 0.877 (95% CI: 0.839~0.916), 0.888 (95% CI: 0.843~0.932), 0.738 (95% CI: 0.675~0.801), respectively. The AUC of class 4a, 4b, 4c and 5 as the cutoff value in the comprehensive diagnostic method of U-M-GI-RADS combined with tumor biomarkers results was 0.877 (95% CI: 0.839~0.916), 0.888 (95% CI: 0.843~0.932), 0.851 (95% CI: 0.799~0.903) and 0.725 (95% CI: 0.661~0.788), respectively. There was no significant difference in diagnostic efficiency between the two comprehensive diagnostic methods. With 4b as the truncation value, the diagnostic efficiency was the highest in the two comprehensive diagnostic methods (Table S2).

The diagnostic efficacy of U-T-GI-RADS or U-M-GI-RADS combined with tumor biomarker results from the comprehensive diagnostic method was slightly lower than that of the single ultrasound method when the 4b, 4c or 5 were used as cut-off values. However, if 4a was taken as the cut-off value, the diagnostic efficiency of the comprehensive method was significantly higher than that of the single ultrasonic GI-RADS diagnosis method.

## 4. Discussion

At present, the transvaginal scan (TVS) is the most commonly used and effective approach for detecting and diagnosing uterine cavity and endometrial lesions. However, the subjective and inconsistent description of ultrasonic interpretation makes its practical clinical application somewhat untrustworthy. The unified definition and description of the IETA expert consensus on the uterine cavity and endometrial diseases formulated in 2010 was aimed at standardizing and improving the level and standardization of reporting. However, the IETA expert consensus has not been widely implemented in the world, especially in our country. In addition, there was no structured diagnostic classification of GI-RADS or O-RADS for uterine cavity and endometrial lesions as there was for adnexal masses. In this study, we pioneered U-T-GI-RADS and U-M-GI-RADS classification methods for uterine cavity and endometrial lesions, both of which had high diagnostic efficacy. This self-generated GI-RADS analysis system of the uterine cavity or endometrial lesions offered an interpretation method to decrease ambiguity and recommend management guides according to its classification. Meanwhile, tumor biomarker results were combined to further improve the diagnostic sensitivity of endometrial cancer and achieve early diagnosis in our study.

The lexicon of GI-RADS for AM was aimed to offer a unified language for the ultrasonic report and to prevent misinterpretation in communication between the sonographer and the gynecologist [16]. The GI-RADS for AM was first described by Amor et al. [45] in 2009. They defined categories 1 to 3 as benign and categories 4 to 5 as malignant; the sensitivity, specificity, PPV, NPV and accuracy for GI-RADS were 92%, 97%, 85%, 99% and 96%, respectively. They concluded that the scoring system could be a good diagnostic method to improve communication between radiologists and clinicians and facilitate the decision-making process [14].

In our study, we also pioneered the U-T-GI-RADS and U-M-GI-RADS classification to evaluate the uterine cavity and endometrial lesions. Our results showed that the U-T-GI-RADS and U-M-GI-RADS classification evaluation methods also had high diagnostic efficiency for uterine cavity and endometrial lesions. 

In our study, there was a statistical difference in partial ultrasound features (e.g., non-uniform endometrial echogenicity: heterogeneous without cysts, endometrial midline appearance: non-linear and no “bright edge” sign) between benign and malignant lesions, but it was difficult to judge benign and malignant lesions based on these ultrasonic indications in practical work because it occupied a large proportion in both benign and malignant lesions. Therefore, we classified these partial ultrasonic signs as undefined signs.

The submucous myomas protruded from the myometrium to the uterine cavity, often resulting in interrupted echo at the endometrial–myometrial junction; it would be overestimated by ultrasound GI-RADS scoring system, resulting in false positives. We could distinguish these lesions from EC by combining the “circular vessels” vascular pattern (in this study, the pathological results of all cases with the circular vessels vascular pattern were submucosal myomas), the characteristics of hypoechoic lesions and pseudocapsule sign (EC were often hyperechoic or mixed echoic, and their boundaries were often unclear) [29]. However, in this study, we also found that EC lesions of the non-endometrioid adenocarcinoma type may also be hypoechoic, quasi-circular in shape, and had scarce blood flow, which may be misdiagnosed as submucous myomas. Therefore, the main distinguishing points of ultrasonic signs are whether the boundary is clear, whether there is a false envelope sign and whether there is a“circular vessels” vascular pattern. Moreover, the tumor biomarker values tend to be higher in EC of non-endometrioid adenocarcinoma types.

The study by Koneczny et al. [43] showed that the GI-RADS classification of adnexal neoplasms produced a high false positive rate (nearly 24.5%). We concluded that the reason for this may be that there is no subdivision of the four categories. In this study, we subdivided four categories between the GI-RADS classification of the uterine cavity and endometrial lesions, and the false positive rates of U-T-GI-RADS classification 4a and 4b were 34.8% and 8%, respectively; the false positive rates of U-M-GI-RADS classification 4a, 4b and 4c were 34.8%, 7.7%, and 1.3%, respectively. In this study, the minimum false positive rate was reduced to 1.3% after subdividing the four categories of lesions.

The endometrial thickness tended to be thicker in endometrial simple hyperplasia cases, and the endometrial midline appearance was often the “not defined” type. Therefore, the two ultrasonic GI-RADS classification methods were prone to over-estimating, and 57.4% (27/47) of endometrial hyperplasia cases were rated as 4a or above. However, the tumor biomarkers values were usually normal (0 cases of endometrial simple hyperplasia with more than two values were positive among HE4, CA125, CA15-3 and CA19-9. Combined with the comprehensive judgment of tumor biomarker results, it could correct the diagnosis of GI-RADS classification and improve the accuracy of diagnosis.

The GI-RADS classification system is promising and reliable for improving the diagnostic ability of ultrasound in malignant lesions of the uterine cavity or endometrium. This is an effective way to solve the conflicts among sonographers with different working experiences. It is a trend to combine the GI-RADS classification system with other tests with the development of new diagnostic instruments [44].

Migda et al. [46] reported that the combination of CA-125 measurements and GI-RADS could significantly enhance the value of diagnostic parameters, such as sensitivity (66.0%), specificity (93.8%), PPV (77.8%), NPV (89.4%), ACC (87.0%) and OR (29.6, CI 12.6–69.6), respectively. The study of Lycke et al. [47] showed that the sensitivity of CA125 and HE4 (>70 pmol/ L) was 96% and 83%, and the specificity was 60% and 91%, respectively, in premenopausal women. In postmenopausal women, the sensitivity and specificity of CA-125 and HE4 (>140 pmol/ L) were 92% and 72%, and 80% and 92%, respectively. Their results revealed that CA125 was a better tumor biomarker for ovarian cancer than HE4. However, we found that the diagnostic efficacy of tumor biomarker results for uterine cavity and endometrial lesions was not consistent with that of adnexal masses. The diagnostic efficacy of HE4 (AUC: 0.628) was the highest, followed by CA19-9 (AUC:0.618), CA125 (AUC: 0.566) and CA15-3 (AUC: 0.513). However, the diagnostic efficacy of these four tumor biomarkers was generally poor. Our results were similar to those of Li et al. [33], Ge et al. [2] and Bian et al. [48]. Among 497 enrolled cases, 33 cases (6.6%) were accompanied by at least two positive values of CA125, CA15-3, CA19-9 and HE4. Eight cases (2.1%) were benign lesions, including three cases of submucous myomas, four cases of endometrial polyps and one case of endometrial polypoid hyperplasia. Twenty-five cases (22.9%) were malignant, including twenty-one cases of endometrioid adenocarcinoma and four cases of non-endometrioid adenocarcinoma. The single diagnostic method of tumor biomarker measurement was not effective in the diagnosis of the uterine cavity and endometrial lesions. However, in this study, most of the patients with two or more tumor biomarkers increased were endometrial cancer patients, so the combined diagnosis was helpful to improve the sensitivity and accuracy of the diagnosis of malignant lesions, especially the cases classified between 3 and 4a and difficult to distinguish between benign and malignant lesions. 

In this study, regardless of which of the four diagnostic methods were used (U-T-GI-RADS, U-M-GI-RADS, U-T-GI-RADS combined tumor biomarkers and U-M-GI-RADS combined tumor biomarkers), if 4b was used as the cut-off value of benign and malignant, and the diagnostic efficiency is the highest. Although the diagnostic efficacy of the two combined methods was similar to or lower than that of the single ultrasound GI-RADS method, the diagnostic efficacy of the two combined methods was significantly higher than that of the single ultrasound GI-RADS method for the lesions classified in class 4a. In clinical work, the most confusing thing is the differentiation of benign and malignant lesions in class 3–4a. The method that can significantly improve the diagnostic efficiency of class 4a lesions and greatly improve our diagnostic confidence is exactly what we are looking for.

## 5. Limitations

There were several limitations in this research. First of all, this study involved only a few hospitals, not a large multi-center study. Second, all examinations were performed by highly experienced sonographers. The repeatability and consistency tests were not performed among physicians with different experiences. Third, most EC patients are endometrioid adenocarcinoma, the number of malignant endometrial lesions with other pathological results was small, and the data were biased. Finally, the GI-RADS classification of the uterine cavity and endometrial lesions was only established by us; it was still uncommon and unfamiliar to many clinicians. Our findings still required verification in extensive clinical practice. Therefore, large-scale and multicenter studies are required in the future.

## 6. Conclusions

Experienced gynecological ultrasound specialists are still scarce, whether in China or other underdeveloped countries. A structured ultrasound reporting system is required to help non-expert operators to more efficiently discriminate between benign and malignant uterine cavity and endometrial lesions. In this study, we pioneered U-T-GI-RADS and U-M-GI-RADS classification methods for uterine cavity and endometrial lesions, both of which had high diagnostic efficacy. The GI-RADS is a pattern recognition classification report that decreases errors in important data in ultrasound reports, reduces inconsistencies in the interpretation of uterine and endometrial lesions and optimizes patient management by standardizing the content and structure of the report. In addition, the systematic use of the GI-RADS system will help monitor manifestation, quality control and possible patient outcomes in the future.

The GI-RADS classification had a good performance in discriminating endometrial cancer. The additional measurement of serum tumor biomarkers CA125, CA19-9, CA15-3 and HE4 improved the diagnostic efficacy for preoperative endometrial carcinoma assessment.

## Figures and Tables

**Table 1:** Univariate analysis of IETA ultrasonic signs in differentiating benign and malignant uterine cavity and endometrial lesions.

Ultrasound Characteristics		Histopathology			
		Benign Lesions	Malignant Lesions	χ2	*p* Value
Endometrial thickness (mm)		9.91 ± 4.13	18.84± 9.21		0.000 *
	Premenopause (mean ± SD)	10.01 ± 4.04	18.02 ± 9.23		0.000 *
	Postmenopause (mean ± SD)	8.89 ± 4.81	19.52 ± 9.21		0.000 *
Uniform endometrial echogenicity		28/388 (7.2%)	1/109 (0.9%)	6.217	0.010 *
	Homogeneous hyperechoic	17/388 (4.4%)	1/109 (0.9%)		
	Homogeneous hypoechoic	0	0		
	Homogeneous isoechoic	2/388 (0.5%)	0		
	Three-layer pattern	9/388 (2.3%)	0		
Non-uniform endometrial echogenicity		360/388 (92.8%)	108/109 (99.1%)		
	Homogeneous with regular cysts	19/388 (4.9%)	0/109 (0%)	5.550	0.019 *
	Homogeneous with irregular cysts	2/388 (0.5%)	0/109 (0%)	0.564	1.000
	Heterogeneous without cysts	305/388 (78.6%)	61/109 (55.9%)	22.480	0.000 *
	Heterogeneous with regular cysts	9/388 (2.3%)	3/109 (2.8%)	0.068	0.795
	Heterogeneous with irregular cysts	25/388 (6.4%)	44/109 (40.4%)	81.908	0.000 *
Endometrial midline appearance					
	Linear	124/388 (31.9%)	3/109 (2.7%)	38.885	0.000 *
	Non-linear	68/388 (17.5%)	4/109 (3.7%)	13.188	0.000 *
	Irregular	91/388 (23.5%)	22/109 (20.2%)	0.518	0.472
	Not defined	105/388 (27.1%)	80/109 (73.4%)	78.174	0.000 *
Endometrial–myometrial junction					
	Regular	368/388 (94.8%)	17/109 (15.6%)	306.143	0.000 *
	Irregular	1/388 (0.3%)	0/109 (0%)	0.282	1.000
	Interrupted	14/388 (3.6%)	66/109 (60.6%)	208.036	0.000 *
	Not defined	5/388 (1.3%)	26/109 (23.9%)	74.083	0.000 *
“Bright edge”				53.137	0.000 *
	Yes	146/388 (37.6%)	2/109 (1.8%)		
	No	242/388 (62.4%)	107/109 (98.2%)		
Intracavitary fluid					
	No fluid	359/388 (92.5%)	70/109 (64.2%)	57.729	0.000 *
	Anechoic or of low-level echogenicity	26/388 (6.7%)	9/109(8.3%)	0.315	0.575
	Ground glass	2/388 (0.5%)	8/109 (7.3%)	20.098	0.000 *
	“Mixed” echogenicity	1/388 (0.3%)	22/109 (20.2%)	76.549	0.000 *
Color score					
	1 point	116/388 (29.9%)	1/109 (0.9%)	39.703	0.000 *
	2 points	264/388 (68.0%)	31/109 (28.4%)	55.316	0.000 *
	3 points	8/388 (2.1%)	45/109 (41.3%)	138.130	0.000 *
	4 points	0/388 (0%)	32/109 (29.4%)	121.747	0.000 *
Vascular pattern					
	No flow	116/388 (29.9%)	1/109 (0.9%)	39.703	0.000 *
	Single vessel (Without branching)	144/388 (37.1%)	1/109 (0.9%)	53.954	0.000 *
	Single vessel (With branching)	14/388 (3.6%)	8/109 (7.3%)	2.800	0.112
	Scattered vessels	86/388 (22.2%)	27/109 (24.8%)	0.329	0.566
	Circular vessels	24/388 (6.2%)	0/109 (0%)	7.084	0.008 *
	Multiple vessels (focal origin)	1/388 (0.3%)	35/109 (32.1%)	128.497	0.000 *
	Multiple vessels (multifocal origin)	3/388 (0.8%)	37/109 (33.9%)	126.525	0.000 *

* represents statistical difference between display rates (*p <* 0.05).

**Table 2:** Multivariate logistic regression analysis of malignant ultrasonographic signs of uterine cavity and endometrial lesions.

Ultrasound Characteristics		B	S.E.	Wald	*p* Value	Exp(B)	95% CI	
							Lower	Upper
Endometrial thickness		0.246	0.026	89.186	0.000 *	1.279	1.215	1.346
	Premenopause	0.252	0.036	48.868	0.000 *	1.287	1.199	1.381
	Postmenopause	0.235	0.052	20.452	0.000 *	1.266	1.143	1.401
Non-uniform endometrial echogenicity								
	Heterogeneous with irregular cysts	2.436	0.283	74.184	0.000 *	11.426	6.564	19.889
Endometrial midline appearance								
	Not defined	2.006	0.245	67.037	0.000 *	7.435	4.600	12.019
Endometrial–myometrial junction								
	Interrupted or Not defined	4.655	0.354	173.299	0.000 *	105.102	52.556	210.184
“Bright edge”								
	No	3.474	0.721	23.199	0.000 *	32.277	7.850	132.707
Intracavitary fluid								
	Ground glass or “Mixed” echogenicity	3.977	0.617	41.595	0.000 *	53.333	15.928	178.579
Color score	3~4 points	4.783	0.416	132.484	0.000 *	119.516	52.927	269.881
Vascular pattern	Multiple vessels (focal origin) or Multiple vessels (multifocal origin)	5.230	0.542	93.193	0.000 *	186.811	64.601	540.209

S.E.: standard error; 95% CI: 95% confidential interval; * The difference was statistically significant (*p* < 0.05).

**Table 3:** The reference table for classification of benign and malignant IETA ultrasonographic features.

Ultrasound Characteristics	Benign Signs	Malignant Signs	Undefined Signs
Endometrial thickness	≤4.0 mm (LR− < 0.1)	Premenopause ≥ 18.5 mm (LR+ > 10), Postmenopause ≥ 15.5 mm (LR+ > 10)	
Uniform endometrial echogenicity	Homogeneous hyperechoic; Homogeneous hypoechoic; Homogeneous isoechoic; Three-layer pattern		
Non-uniform endometrial echogenicity	Homogeneous with regular cysts	Heterogeneous with irregular cysts	Homogeneous with irregular cysts; Heterogeneous without cysts; Heterogeneous with regular cysts
Endometrial midline appearance	Linear	Not defined	Non-linear Irregular
Endometrial–myometrial junction	Regular	Interrupted; Not defined	Irregular
“Bright edge”	Yes		No
Intracavitary fluid		Ground glass; “Mixed” echogenicity	No fluid Anechoic or of low-level echogenicity;
Color score	1~2 points	3~4 points	
Vascular pattern	No flow; Single vessel (Without branching); Circular vessels	Multiple vessels (focal origin); Multiple vessels (multifocal origin)	Single vessel (With branching); Scattered vessels;

LR+: positive likelihood ratio, PLR; LR−: Negative likelihood ratio, NLR.

**Table 4:** Comparison of traditional and modified GI-RADS grading system.

Classification	U-T-GI-RADS	Standard of Classification	U-M-GI-RADS	Standard of Classification
1	Definite benign	No lesions	Definite benign	No lesions
2	Most likely benign	It fits the benign description, not one of the malignant ones	Most likely benign	It fits the benign description, not one of the malignant ones
3	Probably benign	There are undefined signs, but not malignant ones	Probably benign	There are undefined signs, but not malignant ones
4	Probably malignant		Probably malignant	
	4a	Contains 1 malignant sign	4a	Contains 1 malignant sign
	4b	Contains 2 malignant signs	4b	Contains 2 malignant signs
			4c	Contains 3 malignant signs
5	Most likely malignant	Contains more than or equal to 3 malignant signs	Most likely malignant	Contains more than or equal to 4 malignant signs
6	Pathology confirmed		Pathology confirmed	

U-T-GI-RADS: Traditional ultrasound GI-RADS classification for uterine cavity or endometrial lesions. U-M-GI-RADS: Modified ultrasound GI-RADS classification for uterine cavity or endometrial lesions.

**Table 5:** Demographic and clinical variables of the study group.

Parameter	Benign Lesions	Malignant Lesions	*p* Value
Cases number (n)	388	109	
Premenopause (%)	352/388 (90.7%)	49/109 (45.0%)	0.000 *
Postmenopause (%)	36/388 (9.3%)	60/109 (55.0%)	0.000 *
Age (years, mean ± SD)	38.37 ± 9.27	53.00 ± 11.15	0.000 *
BMI (kg/m^2^)	22.65 ± 3.27	24.62 ± 3.94	0.000 *
Gravidity (mean ± SD)	2.43 ± 1.89	2.59 ± 1.57	0.419
Parity (mean ± SD)	1.36 ± 1.06	1.83 ± 1.18	0.000 *
Abortion (mean ± SD)	1.06 ± 1.38	0.74 ± 1.02	0.029 *
Clinical symptoms			
Irregular menstruation (%)	212/388 (54.6%)	54/109 (49.5%)	0.346
Irregular bleeding of the vagina (%)	90/388 (23.2%)	88/109 (80.7%)	0.000 *
Leucorrhea with blood or contact bleeding (%)	11/388 (2.8%)	10/109 (9.2%)	0.012 *
Hypogastralgia (%)	33/388 (8.5%)	8/109 (7.3%)	0.696
No symptom (%)	111/388 (28.6%)	7/109 (6.4%)	0.000 *

BMI: Body mass index (BMI = weight (kg)/height^2^ (m)); mean ± SD: mean ± standard deviation; * represents statistical difference between display rates (*p* < 0.05).

**Table 6:** The evaluation of the diagnostic efficacy of the traditional and modified GI-RADS grading system in differentiating benign and malignant uterine cavity and endometrial lesions.

Methods	Sensitivity (%)	Specificity (%)	Positive Predictive Value (PPV, %)	Negative Predictive Value (NPV, %)	Diagnostic Accuracy Rate
U-T-GI-RADS					
4a	97.2	65.2	44.0	98.8	72.2
4b	88.1	92.0	75.6	96.5	91.2
5	75.2	98.5	93.2	93.4	93.4
U-T-GI-RADS combined tumor biomarkers					
4a	89.9	85.6	63.6	96.8	86.5
4b	81.7	95.9	84.8	94.9	92.8
5	48.6	99.0	93.0	87.3	87.9
U-M-GI-RADS					
4a	97.2	65.2	44.0	98.8	72.2
4b	88.1	92.3	76.2	96.5	91.3
4c	75.2	98.7	94.3	93.4	93.6
5	66.1	99.7	98.6	91.3	92.4
U-M-GI-RADS combined tumor biomarkers					
4a	89.9	85.6	63.6	96.8	86.5
4b	81.7	95.9	84.8	94.9	92.8
4c	71.6	98.7	94.0	92.5	92.8
5	45.0	100.0	100.0	86.6	87.9

U-T-GI-RADS: traditional ultrasound GI-RADS classification for uterine cavity or endometrial lesions; U-M-GI-RADS: modified ultrasound GI-RADS classification for uterine cavity or endometrial lesions.

## Data Availability

The data presented in this study are available on request from the corresponding author. The data are not publicly available due to privacy or ethical restritions.

## Supplementary Materials

The following supporting information can be downloaded at: https://www.mdpi.com/article/10.3390/cancers14225631/s1, Table S1: The ROC curve analysis values of malignant signs and tumor biomarkers. Table S2: The ROC curve analysis values of each diagnostic method. Table S3: The results of traditional ultrasound GI-RADS classification for uterine or endometrial lesions combined with serum tumor biomarkers. Table S4: The results of modified ultrasound GI-RADS classification for uterine or endometrial lesions combined with serum tumor biomarkers. Table S5: Comparison of pathological results of different GI-RADS classification.
