# Correction: Lin et al. IETA Ultrasonic Features Combined with GI-RADS Classification System and Tumor Biomarkers for Surveillance of Endometrial Carcinoma: An Innovative Study. *Cancers* 2022, *14*, 5631

**DOI:** 10.3390/cancers18142280

**Published:** 2026-07-16

**Authors:** Dongmei Lin, Hui Wang, Lu Liu, Liang Zhao, Jing Chen, Hongyan Tian, Lei Gao, Beibei Wu, Jing Zhang, Xia Guo, Yi Hao

**Affiliations:** 1 Department of Medical Ultrasonics, South China Hospital of Shenzhen University, Shenzhen 518000, China; 2 Department of Medical Ultrasonics, The Seventh Affiliated Hospital of Sun Yat-sen University, Shenzhen 518000, China; 3 Department of Medical Ultrasonics, Dongguan People’s Hospital, Dongguan 523000, China; 4 Shenzhen Key Laboratory of Viral Oncology, Center for Clinical Research and Innovation (CCRI), Shenzhen Hospital, Southern Medical University, Shenzhen 518000, China

**Additional Affiliation**

In the published publication [1], there was an error regarding the affiliation for Dongmei Lin. In addition to the affiliation, the updated affiliation should include: Department of Medical Ultrasonics, The Seventh Affiliated Hospital of Sun Yat-sen University, Shenzhen 518000, China. The affiliation numbers for the remaining co-authors were also updated accordingly to reflect the correct sequential order.

**Text Correction**

There was an error in the original publication (because the authors did not list all the ethical approval documents and did not provide a detailed explanation of the sources of the cases).

The correct text content is listed below:

Four hundred and ninety-seven patients with benign or malignant lesions of the endometrium or uterine cavity who underwent ultrasound and tumor biomarker examination and surgical treatment at The Seventh Affiliated Hospital of Sun Yat-sen University (414 cases) or Shenzhen Hospital of Southern Medical University (76 cases) or South China Hospital of Shenzhen University (this hospital is a newly built one; it didn’t officially open for full services until 30 June 2021; therefore, only 7 cases were included) between January 2017 and December 2021 were enrolled in the study. The retrospective study was approved by the Medical Ethics Committee of the three hospitals (KY-2020-030-01, NYSZYYEC20200029 and 20211126001), and the need for informed consent was waived.

**Error in Institutional Review Board Statement**

There was an error in the Institutional Review Board Statement. The previously published statement was incorrect. The correct version is listed below:

Institutional Review Board Statement: This study has been approved by the Medical Ethics Committee of the three hospitals (KY-2020-030-01, NYSZYYEC20200029 and 20211126001).

**Error in Informed Consent Statement**

There was an error in the Informed Consent Statement. The previously published statement was incorrect. The corrected statement appears here:

This study was a retrospective study, and the need for informed consent was waived.

**Error in Funding**

There was an error in the Funding section. The previously published funding statement was incorrect.

The correct version is listed below:

This study was supported by the National Natural Science Foundation of China (No. 82202262), the General project of Science and Technology Innovation Committee of Shenzhen (JCYJ20190814110207603, JCYJ20190814111801681). The funding parties had no influence on the study design, data collection, analysis or interpretation of the results.

The authors state that the scientific conclusions are unaffected. This correction was approved by the Academic Editor. The original publication has also been updated.
