# Supplementary material for: IETA Ultrasonic Features Combined with GI-RADS Classification System and Tumor Biomarkers for Surveillance of Endometrial Carcinoma: An Innovative Study
Source: Cancers (Basel). 2022 Nov 16;14(22):5631. doi: 10.3390/cancers14225631 (PMC9688181; doi:10.3390/cancers14225631)
Supplement: Supplementary file 1 [file cancers-14-05631-s001.zip › cancers-1969923-supplementary.pdf]

cancers MDPI

Supplementary Files

IETA Ultrasonic Features combined with GI-RADS Classification System and Tumor Biomarkers for Surveillance of Endometrial Carcinoma: An Innovative Study

Dongmei Lin, Hui Wang, Lu Liu, Liang Zhao, Jing Chen, Hongyan Tian, Lei Gao, Beibei Wu, Jing Zhang, Xia Guo and Yi Hao

Table S1. The ROC curve analysis values of malignant signs and tumor biomarkers.

Malignant signs or Tumor biomarkers	AUC	SE	95% CI		*P* value
			Lower Boung	Upper Bound	
Endometrial thickness: Premenopause ≥ 18.5mm ,Postmenopause ≥ 15.5mm	0.826	0.025	0.777	0.874	0.000*
Non-uniform endometrial echogenicity: Heterogeneous with irregular cysts	0.688	0.032	0.625	0.751	0.000*
Endometrial midline appearance: Not defined	0.732	0.028	0.677	0.786	0.000*
Endometrial – myometrial junction: Interrupted or Not defined	0.898	0.021	0.856	0.939	0.000*
Intracavitary fluid: Ground glass or "Mixed" echogenicity	0.643	0.033	0.577	0.709	0.000*
Color score: 3~4 points	0.847	0.027	0.795	0.900	0.000*
Vascular pattern: Multiple vessels (focal origin); Multiple vessels (multifocal origin)	0.825	0.028	0.770	0.881	0.000*
Combination of multiple ultrasonic features	0.962	0.012	0.938	0.986	0.000*
CA125 positive	0.566	0.033	0.502	0.630	0.037*
CA15-3 positive	0.513	0.032	0.450	0.575	0.689
CA19-9 positive	0.618	0.033	0.553	0.683	0.000*
HE4 positive	0.628	0.036	0.558	0.698	0.000*

AUC: the area under the curve；SE: standard error；95% CI : 95% confidential interval; CA125: carbohydrate antigen 125; CA15-3: carbohydrate antigen 15-3；CA19-9: carbohydrate antigen 19-9; HE4: human epididymis protein.

Table S2. The ROC curve analysis values of each diagnostic method.

Diagnostic method	AUC	SE	95% CI		*P* value
			Lower Boung	Upper Bound	
U-T-GI-RADS					
4a	0.812	0.019	0.774	0.850	0.000*
4b	0.900	0.020	0.862	0.939	0.000*
5	0.868	0.025	0.819	0.918	0.000*
U-T-GI-RADS combined tumor biomarkers					
4a	0.877	0.020	0.839	0.916	0.000*
4b	0.888	0.023	0.843	0.932	0.000*
5	0.738	0.032	0.675	0.801	0.000*
U-M-GI-RADS					
4a	0.812	0.019	0.774	0.850	0.000*
4b	0.902	0.020	0.863	0.940	0.000*
4c	0.870	0.025	0.820	0.919	0.000*
5	0.829	0.028	0.774	0.884	0.000*
U-M-GI-RADS combined tumor biomarkers					
4a	0.877	0.002	0.839	0.916	0.000*
4b	0.888	0.023	0.843	0.932	0.000*
4c	0.851	0.027	0.799	0.903	0.000*
5	0.725	0.032	0.661	0.788	0.000*

U-T-GI-RADS : Traditional ultrasound GI-RADS classification for uterine cavity or endometrial lesions; U-M-GI-RADS: Modified ultrasound GI-RADS classification for uterine cavity or endometrial lesions; * The difference was statistically significant ($P < 0.05$).

Table S3. The results of traditional ultrasound GI-RADS classification for uterine or endometrial lesions combined with serum tumor markers.

Histopathology	N	U-T-GI-RADS					More than 2 values were positive among HE4, CA125，CA15-3 and CA19-9
		2	3	4a	4b	5	
Intrauterine adhesions	50	0	42	8	0	0	0
Submucous myomas	35	0	0	19	12	4	3
Endometritis	8	0	4	4	0	0	0
Endometrial polyps	181	0	143	33	4	1	4
Endometrial simple hyperplasia	47	0	20	22	5	0	0
Endometrial complex hyperplasia	11	0	5	5	1	0	0
Endometrial polypoid hyperplasia	56	0	39	14	3	0	1
Endometrial hyperplasia with atypia	9	0	2	4	1	2	0
Endometrioid adenocarcinoma	88	0	1	6	12	69	21
Uterine carcinosarcoma	3	0	0	0	0	3	0
Endometrial low grade squamous epithelial lesion	1	0	0	0	0	1	0
Uterine giant cell type high-grade undifferentiated sarcoma	1	0	0	0	0	1	1
Poorly differentiated endometrioid adenocarcinoma with focal clear cell differentiation	1	0	0	0	0	1	0
Endometrial carcinoma (50% endometrioid adenocarcinoma and 50% mucinous adenocarcinoma)	1	0	0	0	0	1	1
Mixed endometrial carcinoma (endometrioid carcinoma and serous carcinoma)	1	0	0	0	1	0	1
Serous adenocarcinoma of endometrium	1	0	0	0	0	1	0
Endometrial infiltrating adenocarcinoma (80% were highly differentiated endometrioid adenocarcinoma and 20% mucinous adenocarcinoma)	1	0	0	0	0	1	0
Endometrial clear cell carcinoma	1	0	0	0	0	1	1
Endometrial undifferentiated carcinoma	1	0	0	0	0	1	0

U-T-GI-RADS : Traditional ultrasound GI-RADS classification for uterine cavity or endometrial lesions.

Table S4. The results of modified ultrasound GI-RADS classification for uterine or endometrial lesions combined with serum tumor markers.

**Histopathology**	**N**	**U-M-GI-RADS**						**More than 2 values were positive among HE4, CA125，CA15-3 and CA19-9**
		**2**	**3**	**4a**	4b	4c	5	
Intrauterine adhesions	50	0	42	8	0	0	0	0
Submucous myomas	35	0	0	19	12	3	1	3
Endometritis	8	0	4	4	0	0	0	0
Endometrial polyps	181	0	143	33	4	1	0	4
Endometrial simple hyperplasia	47	0	20	22	5	0	0	0
Endometrial complex hyperplasia	11	0	5	5	1	0	0	0
Endometrial polypoid hyperplasia	56	0	39	14	3	0	0	1
Endometrial hyperplasia with atypia	9	0	2	4	1	1	1	0
Endometrioid adenocarcinoma	88	0	1	6	12	9	60	21
Uterine carcinosarcoma	3	0	0	0	0	0	3	0
Endometrial low grade squamous epithelial lesion	1	0	0	0	0	0	1	0
Uterine giant cell type high-grade undifferentiated	1	0	0	0	0	0	1	1
Poorly differentiated endometrioid adenocarcinoma with focal clear cell differentiation	1	0	0	0	0	0	1	0
Endometrial carcinoma (50% endometrioid adenocarcinoma and 50% mucinous adenocarcinoma)	1	0	0	0	0	0	1	1
Mixed endometrial carcinoma (endometrioid carcinoma and serous carcinoma)	1	0	0	0	1	0	0	1
Serous adenocarcinoma of endometrium	1	0	0	0	0	0	1	0
Endometrial infiltrating adenocarcinoma (80% were highly differentiated endometrioid adenocarcinoma and 20% mucinous adenocarcinoma)	1	0	0	0	0	0	1	0
Endometrial clear cell carcinoma	1	0	0	0	0	0	1	1
Endometrial undifferentiated carcinoma	1	0	0	0	0	0	1	0

U-M-GI-RADS : Modified ultrasound GI-RADS classification for uterine cavity or endometrial lesions.

Table S5. Comparison of pathological results of different GI-RADS classification.

GI - RADS classification	Pathology		P value
	Benign lesions	Malignant lesions	
1～3	65.2%（253/388）	2.8%（3/109）	0.000*
4a	27.1%（105/388）	9.2% (10/109)	0.000*
4b	6.4% （25/388）	12.8% (14/109)	0.028*
4c～5	1.3% （5/388）	75.2% (82/109)	0.000*

*represents statistical difference between display rates ($P < 0.05$).
